# Synergies of Radiomics and Transcriptomics in Lung Cancer Diagnosis: A Pilot Study

**DOI:** 10.3390/diagnostics13040738

**Published:** 2023-02-15

**Authors:** Aikaterini Dovrou, Ekaterini Bei, Stelios Sfakianakis, Kostas Marias, Nickolas Papanikolaou, Michalis Zervakis

**Affiliations:** 1 Digital Image and Signal Processing Laboratory, School of Electrical and Computer Engineering (ECE), Technical University of Crete, GR-73100 Chania, Greece; 2 Computational BioMedicine Laboratory, Institute of Computer Science, Foundation for Research and Technology-Hellas, GR-70013 Heraklion, Greece; 3 Department of Electrical and Computer Engineering, Hellenic Mediterranean University, GR-71410 Heraklion, Greece; 4 Computational Clinical Imaging Group, Champalimaud Clinical Centre, Champalimaud Foundation, Avenida Brasilia, 1400-038 Lisbon, Portugal

**Keywords:** lung cancer, radiomics, transcriptomics, radiotranscriptomics, diagnosis, statistical analysis, machine learning, enrichment analysis

## Abstract

Radiotranscriptomics is an emerging field that aims to investigate the relationships between the radiomic features extracted from medical images and gene expression profiles that contribute in the diagnosis, treatment planning, and prognosis of cancer. This study proposes a methodological framework for the investigation of these associations with application on non-small-cell lung cancer (NSCLC). Six publicly available NSCLC datasets with transcriptomics data were used to derive and validate a transcriptomic signature for its ability to differentiate between cancer and non-malignant lung tissue. A publicly available dataset of 24 NSCLC-diagnosed patients, with both transcriptomic and imaging data, was used for the joint radiotranscriptomic analysis. For each patient, 749 Computed Tomography (CT) radiomic features were extracted and the corresponding transcriptomics data were provided through DNA microarrays. The radiomic features were clustered using the iterative K-means algorithm resulting in 77 homogeneous clusters, represented by meta-radiomic features. The most significant differentially expressed genes (DEGs) were selected by performing Significance Analysis of Microarrays (SAM) and 2-fold change. The interactions among the CT imaging features and the selected DEGs were investigated using SAM and a Spearman rank correlation test with a False Discovery Rate (FDR) of 5%, leading to the extraction of 73 DEGs significantly correlated with radiomic features. These genes were used to produce predictive models of the meta-radiomics features, defined as p-metaomics features, by performing Lasso regression. Of the 77 meta-radiomic features, 51 can be modeled in terms of the transcriptomic signature. These significant radiotranscriptomics relationships form a reliable basis to biologically justify the radiomics features extracted from anatomic imaging modalities. Thus, the biological value of these radiomic features was justified via enrichment analysis on their transcriptomics-based regression models, revealing closely associated biological processes and pathways. Overall, the proposed methodological framework provides joint radiotranscriptomics markers and models to support the connection and complementarities between the transcriptome and the phenotype in cancer, as demonstrated in the case of NSCLC.

## 1. Introduction

Lung cancer is a common and aggressive type of cancer in both men and women. The majority of the affected population is aged 70 or over, while a small proportion of subjects (1% or lower) diagnosed with lung cancer is younger than 45 [1]. There are two main forms of lung cancer: small-cell lung cancer (SCLC) and non-small-cell lung cancer (NSCLC). NSCLC is the most common form, accounting for more than 85% of lung cancer cases and constitutes the leading cause of cancer-related deaths [2]. The treatment alternatives and the survival rate of people with lung cancer depend mainly on the stage of the cancer when it is diagnosed. Screening at-risk populations of lung cancer development is suggested by doctors as a vital tool to diagnose the disease at an early stage, when the treatment has more chance of success. The most popular screening tests include Computed Tomography (CT), Magnetic Resonance Imaging (MRI), Positron Emission Tomography (PET), and PET/CT scans, while low-dose CT scan is also widely used as a recommended screening test for lung cancer.

Radiomics is a high-throughput image analysis technique that derives a huge amount of quantitative imaging features. These features reflect the heterogeneity, texture, shape, and size of the tumor. The use of “radiomics” has efficiently supported cancer diagnosis, since imaging features reflect the tumor phenotype and staging [3]. Furthermore, radiomic features aspire to effectively complement the qualitative semantic features that are defined by experienced radiologists, since they may uncover distinct tumor phenotypes that are not visible to the unaided eye. Studies have focused on the extraction of quantitative features from medical images in order to reveal the development and progression of cancer, providing valuable information for clinical diagnosis and treatment planning [4]. However, tumors are characterized by somatic mutations. The genotypic markers from molecular biology reflect many aspects of gene and protein interactions across a variety of cellular processes, with the most widely studied being the transcriptomics forum focusing on gene expressions under different conditions. Hence, the unveiling of the way that genetic alterations affect the cell proliferation, and subsequently the tumor texture and shape, is critical for a deeper understanding of the disease.

Several important studies on early diagnosis markers derived from modalities associated with either the phenotype or the genotype of a disease have been conducted [2]. The field of radiotranscriptomics/radiogenomics aims to investigate the combination of the information captured from the phenotype and the genotype of the tumor. The main aspect of radiotranscriptomics is the discovery of the associations between a patient’s transcriptome with imaging phenotypes to support personalized medicine [5,6,7]. In this respect, recent studies have attempted to increase the sensitivity and specificity in early diagnosis, and further associate the prognostic outcome with combined imaging and genomic markers and significant associations between the radiomic features and gene expression patterns [8]. The majority of the radiogenomic studies in NSCLC focus on the prediction of oncogenic mutations, such as the epidermal growth factor receptor (EGFR) and the Kirsten rat sarcoma viral oncogene homolog (KRAS) mutations, solely from the non-invasive radiomic features [9,10,11,12,13,14]. In lung cancer, associations between imaging features extracted from PET/CT scans and gene expressions [15,16,17], as well as between radiomic features extracted from CT medical images and gene expressions and mutations [13,17,18,19], have been investigated.

In radiomics studies, imaging characteristics are accumulated in a massive set of features, which entail either qualitative and quantitative image descriptors measured within the segmented volume of interest [20] or features “engineered” through advanced AI techniques (deep learning [21]). Such features might be considered as byproducts and manifestations of the genomic variation at the cellular level, which controls the specific disease phenotype and/or response to treatment. In that respect, our working hypothesis examines if radiotranscriptomics correlations could uncover the underlying biology of the imaging features and reveal the signaling pathways to comprehensively characterize the morphological complications of the disease at tissue level.

In this work, a methodological framework for the investigation of the associations between the subcellular biomarkers measured through gene expression profiling and the radiomic features extracted from images is proposed. Through these associations, the radiomic features are biologically justified based on the enrichment analysis of their transcriptomics regression models. To this end, a rigorous marker selection procedure was performed in order to derive reliable transcriptomic markers that form the transcriptomics signature, with discrimination and predictive power, as well as stable radiomics markers that form the meta-radiomics signature, with representative distribution across the population. On the basis of these “single-modality” signatures, a combined analysis was conducted by performing regression analysis of radiomics based on transcriptomics, to generate reliable mappings for cancer associations between the two modalities, forming the p-metaomics features.

The novelty of the current study lies in the rigorous methodological approach for the investigation of the transcriptomics features that are subsequently used for the modeling and biological interpretation of the radiomic features. Hence, the arbitrary radiomic features that describe the phenotype are modeled by significant genes, so that their use can be biologically justified by the transcriptomic substrate of that model.

## 2. Materials and Methods

**Figure 1:**
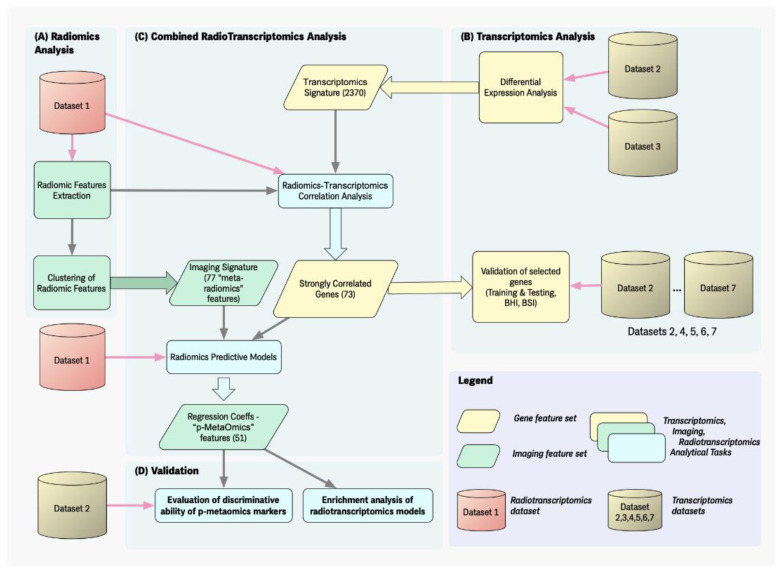
Workflow of the analysis. The overall design of our work can be organized in three main areas: (**A**) In the radiomic analysis, shown on the left, radiomics features are extracted from the imaging datasets and subsequently clustered to yield a limited set of “prototypes” called “meta-radiomics”; (**B**) In the transcriptomic analysis, shown on the right, differential expression analysis produces an initial list of differentiating genes and a transcriptomics-based validation of selected genes is later performed on different gene expression datasets to select the transcriptomics signature; (**C**) In the core of our approach, shown in the central area of the picture, a combined radiotranscriptomics analysis aims to fuse the information discovered in the previous steps, i.e., integrate the radiomics prototypes with the most discriminating gene transcripts and generate a list of “p-metaomics” features. Finally, in (**D**), the discriminative ability of the derived p-metaomics features is evaluated and the radiotranscriptomics associations are biologically interpreted by characterizing the radiomics features based on the biological analysis of their transcriptomics regression models.

The overall design and methodology used is depicted in Figure 1. Three main fields of development, based on the data modalities and the research question, are considered. First, in the radiomics analysis (A), medical imaging data are used to produce an initial number of discriminating radiomics features and then cluster them. In the transcriptomics analysis (B), the most significant (discriminant) genes are identified and validated for their diagnostic potential in NSCLC. This procedure is implemented using statistical analysis and machine learning algorithms for validation of the predictive ability of the derived transcriptomic signature. Third, in the radiotranscriptomics analysis (C), which forms the main contribution of our work, both the imaging and the transcriptomics data are used to investigate joint (radiomics–transcriptomics) predictive associations in NSCLC cancer. Furthermore, the radiotranscriptomics associations are strengthened by exploring the biological justification of radiomics features based on the transcriptomics regression models (D).

Most of the analysis was performed using MATLAB (Mathworks Inc., Natick, MA, USA, version R2016b) with the exception of specific cases, stated in the text, where R (version 4.1.2) and Python (version 3.8.2) programming languages were used.

### 2.1. Datasets

Seven different datasets were used during the procedure analysis. The gene expression microarray data of all datasets were obtained from the publicly available Gene Expression Omnibus (GEO) database. In each dataset, the probesets were coded into their corresponding Entrez Gene ID according to the Illumina platform and, in cases of multiple mappings, the probeset with the largest gene expression value was used to express the corresponding Entrez Gene ID. Table 1 represents an overview of the used datasets. Detailed information regarding the clinical characteristics of used samples is provided in Supplementary Table S1b–h.

#### 2.1.1. Radiomics Dataset

The main dataset is dataset GSE28827 [15,17,22,23] (abbreviated Dataset 1), which contains gene expression microarray data and CT radiomic feature values for 26 patients with NSCLC. This dataset contains gene expression profiles for 24,371 different genes mapped to the Entrez Gene terminology. Furthermore, it contains CT scans for each patient, which are obtained from the publicly available Cancer Imaging Archive (TCIA) database [24].

Using the opensource Pyradiomics (version 3.0.1) Python package and the 3-D Region Of Interest (ROI) of the tumor of the scans, 749 radiomics features were extracted from 24 patients to quantitatively characterize the tumor region. Scans for 2 patients were excluded because the 3D ROI was smaller than 10 pixels. In order to efficiently process the images, several filters were used: the Laplacian of Gaussian, Wavelet, Square, Square Root, Logarithm, Exponential, and Gradient, and radiomics features were calculated for each filtered and unfiltered image. The features represent quantitative data and were classified in seven feature classes in compliance with the definitions introduced by the Imaging Biomarker Standardization Initiative (IBSI) [20]; they are first-order statistics, shape descriptors, texture classes Gray Level Co-occurrence Matrix (GLCM), Gray Level Run Length Matrix (GLRLM), Gray Level Size Zone Matrix (GLSZM), Neighboring Gray Tone Difference Matrix (NGTDM), and Gray Level Dependence Matrix (GLDM) [25].

#### 2.1.2. Transcriptomics Datasets

Since our aim was to methodologically unravel the “radiotranscriptomics” interplay, we initially preferred to explore the above question based on the well-established microarrays transcriptomics data, which have provided researchers with a wealth of biological information and continue to enhance our understanding of disease etiology and pathogenesis, and progress in diagnosis and treatment. Six datasets that contain purely microarray transcriptomics data were used in our analysis. Dataset GSE75037 (abbreviated Dataset 2) [26] entails only gene expression data for 83 patients with matched malignant and non-malignant tissue, resulting in 166 samples in total. Dataset GSE76925 (abbreviated Dataset 3) [27] was included as a control dataset, using only the gene expression data from 40 normal samples in the analysis. Another dataset, GSE18842 (abbreviated Dataset 4) [28], was used for the validation procedure and includes gene expression data for 44 patients with matched cancer and normal tissue, resulting in 88 samples in total. Two additional independent microarray datasets, dataset GSE27262 (abbreviated Dataset 5) [29,30] and GSE30219 (abbreviated Dataset 6) [31], and one RNA-Seq dataset, dataset GSE40419 (abbreviated Dataset 7) [32], were used to enhance the validation procedure. Dataset 5 contains 25 patients with matched cancer and adjacent non-malignant tissue, while Dataset 6 contains 146 malignant samples and 14 normal samples. Dataset 7 provides RNA-Seq gene expression data for 87 malignant samples and 77 adjacent non-malignant samples.

In our study, seven datasets (Table 1) were used to create an indicative large multicenter dataset and select highly discriminant genes between control and cancer cases, beyond the expected measurement variations. Apart from the used data, some additional adenocarcinoma datasets (including the TCGA dataset (RNA-Seq)) that contain gene expression data with paired cases (tumor and their corresponding non-tumor samples) were used for the biological validation via CANCERTOOL during the gene enrichment analysis (Supplementary Table S1a).

### 2.2. Radiotranscriptomics Feature Extraction

#### 2.2.1. Meta-Radiomics Signature Extraction

The radiomics features were clustered in groups based on the similar patterns of the CT imaging features to produce compact groups, reducing the dimensionality of these features. The main dataset, Dataset 1, which was the only dataset that contained radiomic features, was used. From the overall analysis, 42 imaging features were excluded due to their constant value across all samples, with the values of the 707 remaining radiomic features scaled through standardization to homogenize their range of values.

The iterative K-means algorithm [33] using the squared Euclidean distance metric was applied to group together similar imaging features in an unsupervised manner, with 200 iterations and 10 random restarts to avoid trapping in a local minimum. The number of clusters, K, was chosen from testing a wide range from 2 to 100 for fixing the best value according to the inertia metric, Silhouette score [34], and Davies–Bouldin criterion [35,36]. For each derived cluster, we further assessed the homogeneity score [15,17,18] by averaging all pairwise Spearman correlation coefficients within each cluster. We enforced the requirement that clusters should have a homogeneity score greater than 0.75 in order to consider them adequately homogeneous, showing strong correlation as in similar previous studies [15,17]. Each one of the derived clusters that satisfied the dual check of homogeneity, based on distance and correlation metrics, entangled related features encoded in the compact form of a “meta-radiomics” feature. The total set of these features formed the meta-radiomics signature. More specifically, the meta-radiomics feature for each cluster was represented by the nearest imaging feature to its cluster centroid; if more than one imaging feature reflected the same minimum distance from the cluster centroid, the choice was randomly made. Principal Component Analysis (PCA) was applied to the high-dimensional radiomic features space in order to reduce the dimensions and visualize the clustering results. The first two principal components of the radiomic features that were members of some representative clusters and their corresponding meta-radiomic features were used to visualize the derived clusters.

The clustering was used as a dimensionality reduction technique, as the number of radiomics features used in the subsequent analysis was reduced into the number of derived meta-radiomics features that represent homogeneous clusters. However, the clustering procedure grouped together similar features based on their distribution over the entire population, rather than on their nature with the formation of the medical image. For instance, several characteristics of shape might be grouped together with textural characteristics, demonstrating compactness over their population performance rather than on their conceptual nature. Thus, the concrete meta-radiomics features signify characteristic distributions of radiomics features over the population.

#### 2.2.2. Transcriptomics Signature Extraction

The gene expression values of cancer samples of Dataset 2 and the gene values of normal samples of Dataset 3 were used to identify genes of significant differentiation ability in a set of microarray experiments. The intersection of Dataset 2 and Dataset 3, which consisted of 16,252 common genes, was used for further analysis. However, due to the fact that the cancer and normal samples of each gene were derived from two different datasets, mean-centering normalization was applied as a preprocessing step in both datasets independently to restrict the “batch effects” [37] (e.g., different staff members, platforms, and laboratory conditions) and make the two datasets comparable.

Subsequently, Significance Analysis of Microarrays (SAM) in R (via Shiny package, version 1.7.4) by Tusher et al. [38], with the additional setting of 2-fold change, was used between the mean-centered values of the 16,252 common genes from Dataset 2 and Dataset 3, to identify genes that differed significantly between the two sets of microarray experiments. SAM was performed for the two-class unpaired problem with its “delta” (Δ) parameter being chosen with respect to the criterion of FDR lower than 5%; the induced value of Δ was 3.94. Regarding the number of permutations, Damle et al. [39] showed that as the number of permutations increases, the FDR decreases. Accordingly, the number of permutations was set equal to 1000. In order to strengthen the statistical significance of differential genes and control the batch effects, the criterion of 2-fold change between cancer and normal samples of the same dataset, Dataset 2, was also imposed.

The differentially expressed genes were further examined in terms of their statistical correlation with the radiomic features. Such correlations are considered important for cancer diagnosis in order to investigate the underlying biology and connection of the transcriptome and the phenotype. Dataset 1 was the only dataset that contained both microarray and imaging data for all the patients and it was thus used to investigate significant correlations between the CT imaging features and differentially expressed genes. The 42 radiomic features that had constant values across all samples were excluded from the analysis and thus the remaining 707 features were used for the correlation analysis. The significant correlations between the 707 radiomic features and the DEGs were investigated using two statistical methods: the Spearman rank correlation test and the SAM for quantitative problems.

The non-parametric Spearman rank correlation test was performed between each pair of genes and imaging features, as in similar studies [15,40]. Furthermore, an FDR-controlling statistical approach using the Benjamini–Hochberg procedure across each gene was applied to correct for multiple comparisons and enhance the statistical significance of the derived correlations (FDR threshold: 5%). The Spearman rank correlation test is a rank-based non-parametric method that can handle non-normal data and measures the monotonic relationship between two variables, identifying the most important correlations. In the second method, the values of the differentially expressed genes were imported as input to the SAM and the continuous-valued imaging feature as the response variable, yielding 707 different SAM tests, with one for each imaging feature. All features were transformed into ranks and scaled with the standard deviation in order to use the Spearman rank correlation coefficient as a statistic in SAM’s computations and be consistent with the first method. The number of permutations was set equal to 1000 and the Δ value was chosen in each SAM so that the minimum FDR of 5% was achieved (q-value ≤ 0.05). The DEGs that preserved significant associations with radiomic features with both statistical methods formed the transcriptomic signature with the most significant biomarkers for NSCLC. As a visualization assessment, before being used in further analysis, a heatmap (heatmap.2 function of gplots package in R, version 3.1.1) was implemented to compare the expression profiles of the selected genes on the cancerous and normal samples of Dataset 2 that contains matched malignant and non-malignant lung samples.

In order to assess and validate the predictive ability of the selected genes, the Support Vector Machine (SVM) classifier was performed using multiple kernels (linear, Gaussian, polynomial) to assess its performance; the linear kernel had the best performance. The SVM linear classifier was trained on the samples of Dataset 2 and tested on the external validation Dataset 4. Another validation test for the selected transcriptomic signature was performed by assessing the compactness of genes in each condition of the new Dataset 4. More specifically, the Biological Homogeneity Index (BHI) and the Biological Stability Index (BSI) [41] were calculated in the R programming language as metrics to assess the derived statistical clusters. The BHI and BSI were calculated in two different formulations on the clustered samples of Dataset 4, with hierarchical clustering (hclust function of stats package in R, version 4.1.2) using the expression profiles of the significant genes. In the first test, the BHI investigated if the selected genes indeed produced two biologically homogeneous clusters corresponding to the “cancer” and “normal” conditions. In the second formulation, the BHI was used to assess if the genes of the same biological class were grouped together in the same statistical cluster. The biological class of the genes is considered to be either positive or negative, according to the initial differentially expressed genes analysis with the SAM algorithm. The BSI was calculated to assess the consistency of the statistical clusters, derived using full and reduced data space, with respect to the biological class of the clustered samples.

The transcriptomics signature was extracted and validated for its diagnostic potential using DNA microarray data. Hence, two new DNA microarray datasets (Datasets 5 and 6) and one RNA-Seq dataset (Dataset 7) were utilized in order to evaluate the ability of the transcriptomics signature to discriminate between malignant and non-malignant tissue also on an RNA-Seq dataset. More specifically, the two external datasets with DNA microarray data (Datasets 5 and 6) were used to train an SVM linear classifier and the dataset with RNA-Seq data (Dataset 7) was used as the test set. The aim of this classification schema was to investigate whether the selected genes, which had been derived using their microarray expression profiles, can predict the type of lung tissue using their expression profiles extracted by the RNA-Seq technology, using three unseen datasets.

The DEGs of the transcriptomic signature were extracted using datasets of normal samples and lung adenocarcinoma (LUAD), which is the most common subtype of NSCLC. The main goal was to identify genes that have differentiation ability between malignant and non-malignant samples. In the validation stage, the malignant samples were NSCLC cases derived from patients diagnosed with LUAD, but also with lung squamous cell carcinoma (LUSC), which are the two major subtypes of NSCLC. Thus, datasets that contained both LUAD and LUSC patients were utilized to validate the ability of the selected genes to differentiate between malignant and non-malignant samples, independent of the NSCLC subtype. Overall, the selected genes of the transcriptomic signature were well-validated for their differentiation ability between malignant (NSCLC) and non-malignant samples, even under the variability within the NSCLC samples stemming from the two different subtypes of NSCLC.

#### 2.2.3. p-Metaomics Signature Modeling

The modeling of the radiomic features from the transcriptomics data was investigated using regression analysis. More precisely, we investigated the degree in which the power of radiomic markers can be modeled and approximated by molecular markers, giving a means to replace the actual radiomic measurements with approximate (or simulated) values obtained from regression models built on the basis of genes. Thus, this modeling methodology provides a tool for the formation of artificial imaging features from precursor molecular entities.

The derived gene expression data (i.e., transcriptomics signature) and the radiomic features data (i.e., meta-radiomics signature) from the main Dataset 1 were used in a multiple regression analysis with Lasso regularization [42], in order to model the relationship between the dependent variable, i.e., each meta-radiomics marker, and the independent predictors, i.e., genes in the transcriptomics signature. Lasso regularization was used to select important features while reducing the effects of overfitting, as the number of predictors was larger than the number of observations, with Leave-One-Out Cross Validation (LOOCV) to minimize the cross-validated Mean Squared Error (MSE). The model’s performance was assessed with the R-squared metric (also known as the “coefficient of determination”) and models evaluated with R-squared values above 0.70 were considered to adequately predict the meta-radiomic features. Each one of the predicted meta-radiomics features that satisfied this criterion was termed as a “p-metaomics” feature, since it is constructed from linear combinations of genes of the transcriptomics signature with the identification prefix “p” to imply that it is a prediction.

Notice that p-metaomics correspond to original radiomic features that correlate with the transcriptomic signature, and each p-metaomics feature is predicted from its corresponding model built from a subset of genes with non-zero regression coefficients rather than the entire transcriptomics signature. Additional validity metrics, including the Normalized Root Mean Squared Error (Normalized RMSE), Pearson correlation between the predicted meta-radiomic feature and the actual one, and the Cross Validated Normalized RMSE, were used to assess the performance of the predictive models [43].

### 2.3. p-Metaomics Model Evaluation

The discriminative value of the modeled (or simulated) p-metaomics features, which span a combined space from the gene expression profiles, was explored by utilizing the radiotranscriptomics correlations in approximating the radiomics feature space from the transcriptomic vector space. Dataset 2 contains genes expression profiles from malignant and adjacent non-malignant samples, but it does not provide information on any form of imaging features. Thus, artificial model approximations of p-metaomics markers were produced using the corresponding regression coefficient for each significant gene in the model. To this end, a heatmap (heatmap.2 function of gplots package in R, version 3.1.1) with the values of the cancer and non-malignant samples of the derived p-metaomics signature was implemented to examine the discrimination ability of these simulated features in cancer versus control populations. Furthermore, the BHI and BSI metrics were calculated in R in order to assess the homogeneity of the class separation produced by the p-metaomics models. Thus, the samples of Dataset 2 were clustered based on the values of the p-metaomics features, to assess whether they can produce biologically homogeneous clusters based on the cancer and normal conditions of samples.

### 2.4. Enrichment Analysis

A two-step process was performed to infer biological consequences from the derived gene sets. The first step revealed the enriched terms for each gene set via the WEB-based GEne SeT AnaLysis Toolkit (WebGestalt) [44], whereas the second step evaluated the genes of interest in subgroups of subjects (e.g., nontumoral/tumoral tissue, progression stages, mutation status) in independent well-annotated lung cancer datasets via CANCERTOOL [45].

WebGestalt is a web tool for functional enrichment analysis, used here to extract shared characteristics of derived gene sets at various stages of our radiotranscriptomics analysis. The current version of WebGestalt (2019) was used, and it supports a plethora of functional categories from well-known public databases, such as the: (i) knowledge databases, including the Gene Ontology (GO) database and the Molecular Signatures Database (MSigDB), that contains information for potential targets of regulation by transcription factors (TFs) or microRNAs (miRNAs); (ii) gene pathway databases, including KEGG, Panther, and Wikipathway; (iii) interaction network database BioGRID; and (iv) RNA-Seq database Firehose-Broad GDAC, which contains NSCLC datasets (lung adenocarcinoma (LUAD) and lung squamous cell carcinoma (LUSC) from The Cancer Genome Atlas (TCGA)) [44]. Using over-representation analysis and the statistical correction of Benjamini–Hochberg of WebGestalt (FDR threshold: 5%), the functional terms of the transcriptomic signature and the p-metaomics gene sets were examined in different biological contexts. Thus, biological processes, pathways, and network modules, as well as regulators of gene expression transcription factors (TFs) (acting at the transcriptional level) and miRNAs (acting at the post-transcriptional level), which may be associated with NSCLC, were included. The entire genome was used as a reference set. In order to provide biological terms with high information content, the top 10 enriched terms were considered, which in many cases fulfill the criteria for both low *p*-values and low adjusted-FDR values.

CANCERTOOL is a web-based interface that provides access to transcriptomics cancer data selected for rich clinical annotation. Using statistical analysis tools (Student’s *t*-test or ANOVA, Kaplan–Meier estimator, and Cox model) on the lung adenocarcinoma datasets of CANCERTOOL (lung squamous cell carcinoma data were currently not available), we aimed to compare the relative expression of the derived DEGs of the transcriptomic signature against: (a) tumor versus healthy tissue, (b) different pathologic feature (i.e., stages), (c) molecular characteristics of the tumors (e.g., KRAS and EGFR mutation status), and (d) disease progression (i.e., disease-free/metastasis-free/overall survival) [45].

## 3. Results

### 3.1. Meta-Radiomics Signature

**Figure 2:**
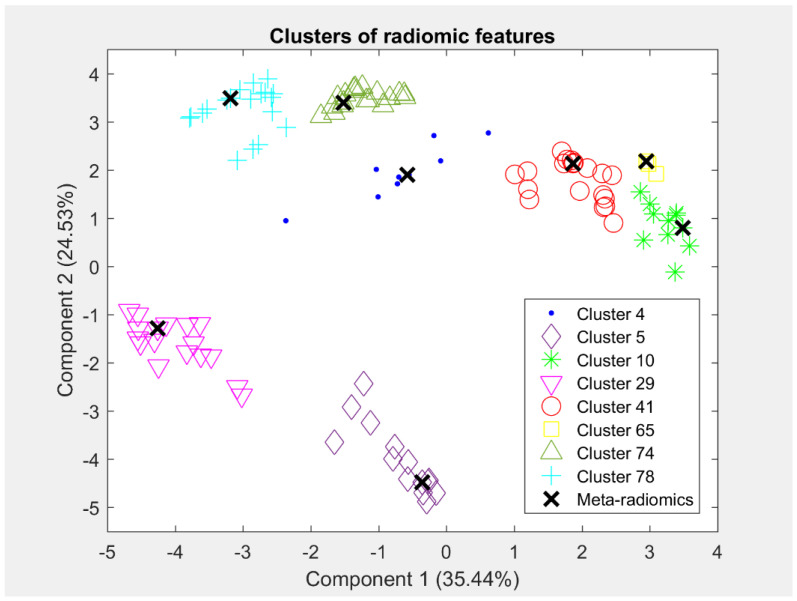
Clusters of radiomic features along with their corresponding meta–radiomic features (i.e., features closest to centroids) using the first two principal components. Each point represents a radiomic feature.

The K-means algorithm produced 95 clusters of co-expressed radiomic features based on the scores in the Silhouette and Davies–Bouldin criteria. More precisely, this K achieved a Silhouette score equal to 0.41 and a Davies–Bouldin index equal to 1.01. Furthermore, the homogeneity score of each of the 95 derived clusters was computed to assess homogeneous radiomic clusters. Thus, 77 of the 95 clusters satisfied the criterion of a homogeneity score greater than 0.75, forming the meta-radiomics signature. Each of the 77 homogeneous clusters (Table S2) consisted of a different number of imaging features, which are globally represented by a single “meta–radiomics” feature, being closer to the centroid of the cluster. Thus, this step produced 77 meta–radiomics markers that express groups of co-expressed imaging features. In total, 600 of the initial 707 imaging features were involved in the 77 homogeneous clusters. In Figure 2, the derived clusters are presented by using the first two principal components of the radiomics members of each cluster, along with its corresponding meta–radiomic feature. The different colors and symbols represent the different groups of co-expressed radiomic features. The names of the radiomics features, which are members of each cluster, are presented in Table S2.

### 3.2. Transcriptomics Signature

The extraction of the transcriptomics signature aims to identify the most discriminant and representative genes in lung cancer to be identified as transcriptomic markers. SAM with 2-fold change identified 7014 significant genes with a q-value equal to 0%, using the gene expression profiles of the cancer samples of Dataset 2 and the normal samples of Dataset 3 (cancer versus control samples). Strengthening the discrimination power and controlling the batch effects by testing samples of the same dataset, 2415 of the 7014 genes remained significant according to the 2-fold change within Dataset 2 (cancer versus control). Projecting to Dataset 1, 2370 significant genes were identified, with 1540 being positive (i.e., higher values in cancerous than normal samples) and 830 being negative (i.e., higher values in normal than cancerous samples) significant. Taking the common transcriptomic features that were found to be statistically correlated with radiomic features in both statistical tests (SAM and Spearman rank correlation test with FDR), a set of 78 significant biomarkers for NSCLC that consists of differentially expressed genes, also preserving significant associations with radiomic features, was identified (Supplementary Table S3). The 78 DEGs included 66 over- and 12 under-expressed genes.

**Figure 3:**
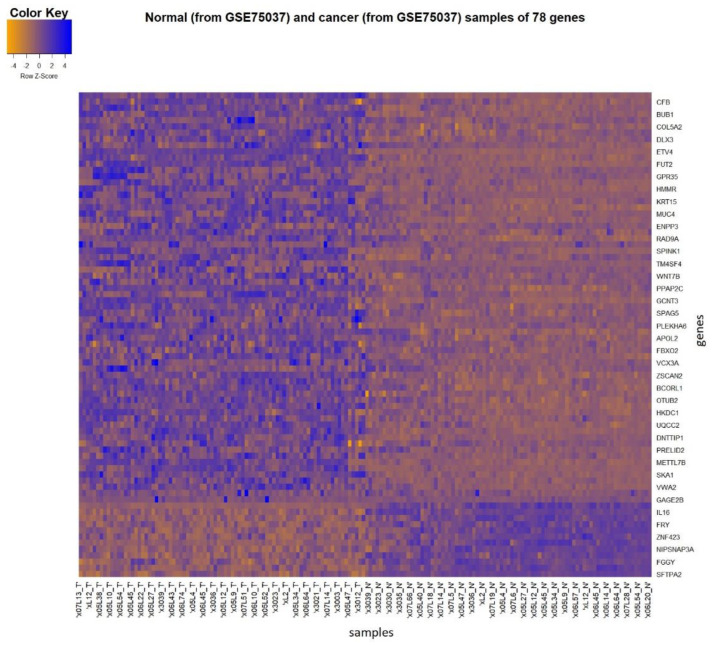
Heatmap for the visualization of the expression profiles of the 78 significant genes using 83 non–malignant and 83 cancer samples from Dataset 2. Scanning from top to bottom, the positive significant genes are displayed first, followed by the negative significant genes. The cancer samples in this case are displayed in the left side of the figure and the normal samples in the right side. Blue color indicates higher values and orange color indicates lower values, as shown in the “color key” bar.

In Figure 3, a heatmap of the selected transcriptomic features is presented in order to visualize the difference in the expression profiles between the cancer and the non-malignant samples. The figure illustrates the different values of the gene expression profiles using a color scale from blue (i.e., higher values) to orange (i.e., lower values). The discrimination of the two gene groups (positive or negative) is reflected in the heatmap. An adequately clear separation of cancer and non-malignant samples is observed based on the expression profiles of the positive and negative significant genes. In the right side of Figure 3, a small distribution disturbance is shown for some genes of control samples. The control samples in Dataset 2 had been derived from adjacent non-malignant tissue. Hence, this small deviation for some genes may be attributed to the special histopathological decomposition of these samples.

**Figure 4:**
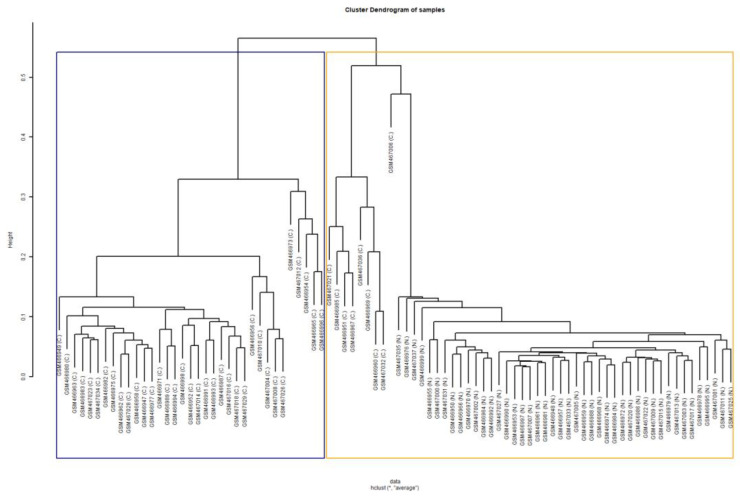
Dendrogram of 88 samples (Dataset 4) derived by their hierarchical clustering. The blue and orange boxes indicate the two produced statistical clusters that contain the largest proportions of the cancerous and normal samples, respectively. The condition of each sample is indicated within the parentheses. At the bottom, the function, i.e., “hclust”, used in R for the implementation of the dendrogram is presented. The “*” in the function indicates the first argument of the function, which is a dissimilarity structure that is stored at the variable “data” and it is used for the implementation of the dendrogram. The second argument indicates the agglomeration method used. Abbreviations: C., cancer; N., normal.

**Figure 5:**
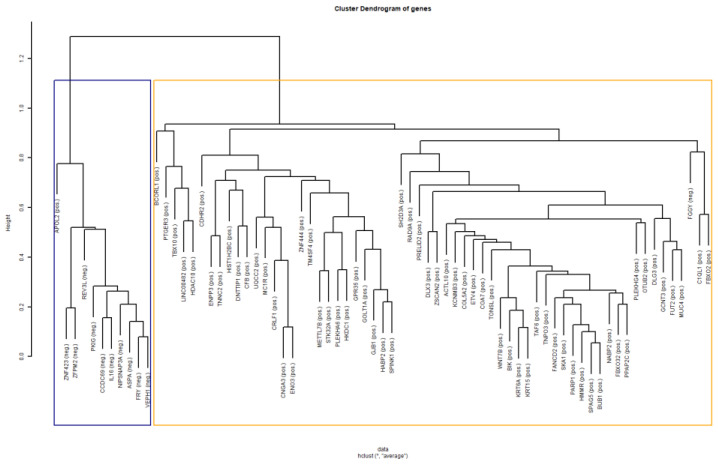
Dendrogram of 73 genes derived by their hierarchical clustering (using Dataset 4). The blue and orange boxes indicate the two produced statistical clusters that contain the largest proportions of the negative and positive genes, respectively. The condition of each gene is indicated within the parentheses. At the bottom, the function, i.e., “hclust”, used in R for the implementation of the dendrogram is presented. The “*” in the function indicates the first argument of the function, which is a dissimilarity structure that is stored at the variable “data” and it is used for the implementation of the dendrogram. The second argument indicates the agglomeration method used. Abbreviations: neg., negative; pos., positive.

The transcriptomics signature was validated with the assessment of the predictive ability of the genes in tissue classification (cancer versus normal tissue), using the external Dataset 4 as the test set. Since, the new Dataset 4 did not contain 5 of the 78 significant genes, these 5 genes were excluded from the analysis. The classifier showed great performance, achieving accuracy of 92.05%, sensitivity of 84.09%, and specificity of almost 100%. The high performance of the classifier, assessing all the validity metrics, indicated that these 73 genes have the added potential to predict or classify cancer or normal samples. Furthermore, the compactness of the genes was assessed using the external Dataset 4. The BHI and BSI of the first test, after performing clustering of samples based on the gene values, were equal to 0.856 and 0.833, respectively, indicating that a large proportion of samples with the same biological label were consistently grouped together into the same statistical class. In Figure 4, the derived dendrogram of samples is presented, showing the members of the two statistical clusters produced by hierarchical clustering of the samples. The majority of the cancerous samples have been grouped together into the blue cluster, while the normal samples and a small proportion of cancer samples have been grouped together into the orange cluster. These clustering results are quantitatively expressed by the values of the BHI and BSI metrics of the first test. Similarly, the outcome of the second formulation corresponding to the clustering of genes resulted in BHI and BSI equal to 0.893 and 0.892, respectively. In Figure 5, the derived dendrogram of genes is presented, showing that the majority of the positive and negative genes have been grouped together into two different statistical clusters, respectively. Only one positive and one negative gene have been misclassified to the opposite cluster, justifying the high values of the metrics. The high value of BHI in both tests implies that the derived genes also provide the power to group together tissue samples of the same biological class. The high value of BSI in both tests confirms the ability of the transcriptomics signature to separate cancer from normal samples by producing consistent biologically homogeneous clusters. Regarding the discrimination ability of the 73 genes to classify the lung tissue type based on RNA-Seq measurements, the classifier that used the three external datasets (Datasets 5, 6, and 7) showed very good performance, achieving an accuracy of 89.02%, sensitivity of 79.31%, and specificity of 100%.

### 3.3. p-Metaomics Signature

The modeling of the p-metaomics markers is based on the linear regression of meta-radiomics markers from the transcriptomic ones. Thus, 53 of the 77 radiomic models satisfy the criterion of R-squared greater than 0.70, revealing that 53 meta-radiomic features can be predicted from genes with an accuracy of 70% or more. The p-metaomics signature consisted of these 53 p-metaomics markers, which engage 449 original radiomic features. The subset of genes that predicts each p-metaomics marker differed over the regression models, but all 73 genes participated in at least one predictive model.

Examining the relationship of the 53 actual meta-radiomics features with their corresponding p-metaomics approximations, the normalized RMSE was almost 0 (ranging from 0.007 to 0.14) and the Pearson correlation coefficient was greater than 0.88, with all the corresponding *p*-values of the Pearson correlation coefficients being significantly less than 1%, indicating strong relationships between actual and predicted meta-radiomics features (Tables S4 and S5). However, after examining the correlations between pairs of the selected p-metaomics and the genes, 2 of the 53 models were found not to be statistically correlated with genes. Thus, these two models were excluded from further analysis, resulting in 51 significant p-metaomics markers that form the p-metaomics signature (Table S5), which, according to the radiomics clustering results, represent 440 radiomic features.

**Figure 6:**
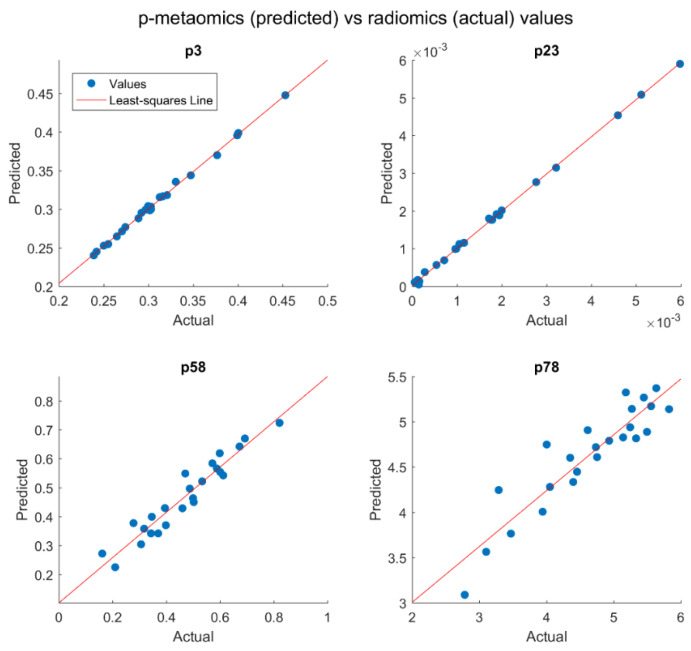
Plots visualizing the actual values against the predicted values of radiomic features. The p–metaomics features (i.e., simulated radiomic features from genes) were derived by multiple linear regression models with Lasso regularization.

In Figure 6, the results of the multiple linear regression models for some meta-radiomic features are presented by plotting the actual values against the predicted values of the radiomic feature. The red line represents the least-squares line for these values to show the linear relationship between the actual and the predicted values of each feature. The predicted values (i.e., p-metaomics feature) should be as close as possible to the actual values. As shown in the first row of Figure 6, the values of the p3 and p23 features are relatively close to the values of the original radiomic feature, as the majority of the points coincide with the line and their regression models achieved R-squared values of 99.7% and 99.9% (Table S5), respectively. In the second row of Figure 6, the p58 and p78 features present linear relationships with the original radiomic features to a lesser extent, as the points scatter slightly above and below the line and their regression models achieved R-squared values of 90.0% and 78.1% (Table S5), respectively.

The p-metaomics features are simulated radiomic features derived by a linear combination of genes. For instance, the p-metaomics feature p78 represents the predicted values of the radiomic feature “wavelet_1_original_glcm_SumEntropy” that was derived with 78.1% accuracy (Table S5) based on the linear combination of 8 genes, namely, *MUC4*, *PTGER3*, *PLEKHA6*, *CDHR2*, *PKIG*, *ZNF423*, *ZFPM2*, and *VEPH1*. Hence, each of these 8 genes was multiplied by the corresponding coefficient, which had been calculated from the training of the regression model. Subsequently, these values were summated to derive the values of the p-metaomics feature p78 that simulates the “wavelet_1_original_glcm_SumEntropy” feature. This radiomic feature was biologically justified via the enrichment analysis performed on the set of these 8 genes.

### 3.4. Evaluation of the Discrimination Ability of p-Metaomics Models

**Figure 7:**
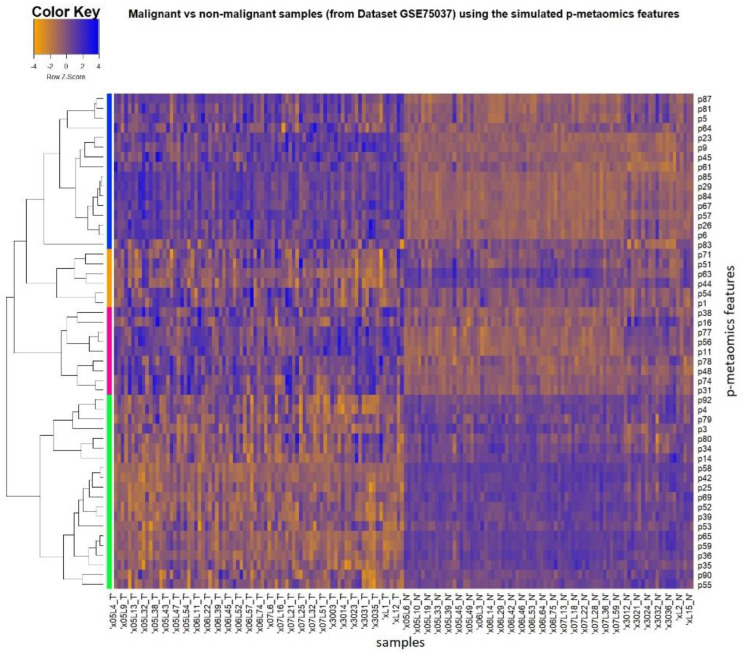
Heatmap visualizing the cancer and normal samples of Dataset 2 using the p–metaomics signature (i.e., simulated radiomics) of 51 features.

Similar to the heatmap in Figure 3 for the entire Dataset 2, Figure 7 provides visualization of level differences between the cancer and control samples for the derived p-metaomics signature of 51 features. The vertical axis depicts the p-metaomics feature vector. The dataset itself does not contain any radiomic features, which are only modeled by transcriptomics as p-metaomics features. The class discrimination is evident, even though the marker set only reflects simulations of imaging characteristics from linear transformations of transcriptomic markers. More specifically, the 83 NSCLC samples of Dataset 2 are displayed in the left side of the figure, while the 83 non-malignant samples are presented in the right side. The upregulation (blue values) and the downregulation (orange values) regions of the p-metaomics models between the cancer and the non-malignant samples are clearly distinguished, showing the discrimination between these two groups of samples (cancer versus non-malignant). However, in the right side of the figure, there is a small portion of non-malignant samples that deviates from the homogeneous distribution of the rest of the non-malignant samples for some p-metaomics models. The p-metaomics models are linear regression models, introducing a margin of error in estimation. These samples present the largest error in the proposed modeling of p-metaomics features. Furthermore, these samples are the same as those that produced the deviation from the homogeneous distribution in the heatmap of Figure 3, in which the differentially expressed genes were used. Thus, Figure 7 also reveals samples that could possibly be further examined for their histopathological decomposition. The dendrogram on the *y*-axis reveals the four dominant groups of p-metaomics features that are over- and under-expressed. The two groups of p-metaomics features in each category (i.e., over- and under-expressed) indicate the different degree of their differentiation ability. For instance, the green group of under-expressed p-metaomic features can more accurately discriminate between malignant and non-malignant samples than the corresponding orange group, as shown in Figure 7. Furthermore, the BHI and BSI were equal to 0.752 and 0.821, respectively, indicating that the p-metaomics models can also separate the cancer from normal samples, producing adequately biological homogeneous clusters. The values of the BHI and BSI metrics objectively validate the discrimination ability of the p-metaomics features, which was visually presented in the heatmap. The p-metaomics models are derived by linear combinations of genes, which have been rigorously validated for their discrimination ability. Hence, these modeled features retain the ability of their transcriptomic substrate to discriminate between malignant and non-malignant samples, conveying information regarding cancer-related factors.

### 3.5. Enrichment Analysis of Radiotranscriptomics Models

#### 3.5.1. Overrepresentation Analysis on DEGs

The top 10 enriched GO biological processes terms include nucleoside triphosphate metabolic process, digestion, acute inflammatory response, carbohydrate catabolic process, and response to radiation, as shown in Supplementary Table S6a. KEGG and Panther analysis revealed that those 73 genes were enriched in several cancer-related metabolic pathways, including glycolysis/gluconeogenesis and pentose phosphate pathway [46]. Wikipathway analysis showed enrichment in specific signaling pathways, such as ATR/ATM Signaling, and DNA IR-Double Strand Breaks (DSBs) and cellular response via ATM (Table S6b), the latter of which reached statistical significance. Network analysis by WebGestalt revealed four statistically significant enriched co-expression modules in lung adenocarcinoma data (LUAD-M722, LUAD-M919, LUAD-M375, LUAD-M65) and one protein-interaction module (BIOGRID-M698) (Table S6c). The identified enriched GO biological processes associated with these modules correspond to digestive system process, glycosphingolipid metabolic process, xenobiotic metabolic process, regionalization, and mitochondrial genome maintenance, respectively. In addition, several transcription factors’ binding sites were found enriched, including known sites for Hepatic Nuclear Factor (HNF1) and Androgen Receptor (AR) that reached a favorable statistical trend (FDR = 0.08). Finally, the top 10 enriched predicted miRNAs that targeted the DEGs, such as miR-143, miR-29A, miR-29B, and miR-29C, are shown in Table S6d.

According to the enrichment analysis of the 73 DEGs in NSCLC, the DNA IR-Double Strand Breaks (DSBs) and cellular response via ATM was the most important finding. In our study, the genes involved in this pathway were *PARP1*, *FANCD2*, *RAD9A*, and *NABP2*, which operate via the ataxia-telangiectasia mutated (ATM) gene. Although DSBs occur normally during DNA replication, meiosis, and immune system development, they are the most hazardous lesions arising in the genome of eukaryotic organisms, and their efficient repair is crucial in maintaining genomic integrity, cellular viability, and the prevention of tumorigenesis. The ATM gene is critical in maintaining genomic integrity and plays a key role in the cellular DNA damage response. In response to DNA double-strand breaks, ATM phosphorylates downstream proteins involved in cell-cycle checkpoint arrest, DNA repair, and apoptosis [47].

#### 3.5.2. Biological Interpretation of Radiomics via the p-Metaomics Models

**Figure 8:**
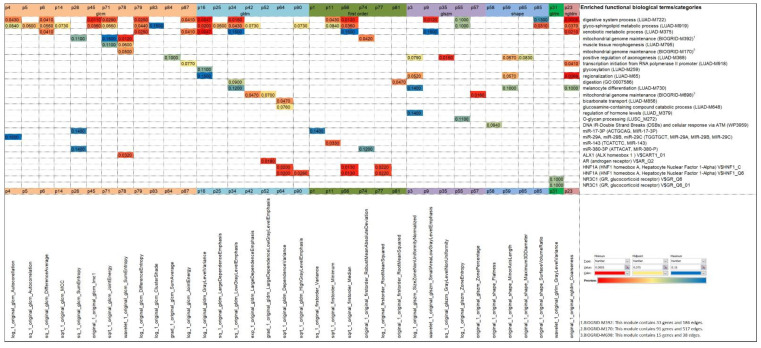
Overview of the enriched functional biological terms/categories resulting from the overrepresentation analysis of the gene sets of p-metaomics models. FDR was set to ≤0.1. Statistically significant terms are highlighted in deep red.

In order to establish a causal relationship between radiomics (phenotype) and gene expression (intermediate between genotype and phenotype), the regression models of radiomics based on transcriptomics were exploited to characterize the GO biological processes, pathways, network modules, and TF- and miRNA-targets, using the online tool WebGestalt. The most significant associations of radiomics markers with functional biological terms, as revealed by the gene sets in the p-metaomics models, are summarized in Figure 8. According to overrepresentation analysis, we observed that the gene sets of 25 p-metaomics models included statistically significant enriched terms (FDR ≤ 0.05) and the gene sets of 11 p-metaomics included relatively significant enriched terms (0.05 < FDR ≤ 0.1) (Table S7).

Since the gene sets of regression models stem from the same 73 gene signature, we would expect to find similar enriched terms as above, with some degree of variation in their ranking. Indeed, we found several same enriched terms that remained significant (e.g., LUAD-M919, LUAD-M722, transcription factors’ binding sites for HNF1, miR-143) or reached a slightly lower significance level (e.g., DNA IR-Double Strand Breaks and cellular response via ATM), but mainly a higher significance level in one or more p-metaomics models (e.g., GO:0007586; LUAD-M856, LUSC-M272, BIOGRID-M392, MIR-29A, MIR-29B, MIR-29C) (Figure 8). Furthermore, additional enriched terms emerged in several p-metaomics models, such as LUAD-M259, LUAD-M379, LUAD-730, and BIOGRID-M170.

Focusing on the 36 most significant p-metaomics models (FDR ≤ 0.1) (as illustrated in Figure 8 and Table S7), several observations were made. Each gene set is governed either by one or a unique mixture of biological processes and/or TFs/miRNAs. More specifically, 23 p-metaomics models harbor more than one significant functional term from the same or a different functional category. Furthermore, several gene sets of p-metaomics models are related to specific biological processes, such as the p78, p64, p3, and p11 features associated with the mitochondrial genome maintenance (BIOGRID-M170), the bicarbonate transport (LUAD-M856), the regulation of hormone levels (LUAD-M379), and the miR-143, respectively. However, many gene sets of cluster centroids are enriched by closely related processes, such as the enriched O-glycan processing, glucosamine-containing compound catabolic process, and glycosylation in the p55-, p64-, and p16-metaomics models, respectively. Moreover, some gene sets of cluster centroids are enriched by closely related modules or predictive TF motifs that are linked to unique processes or TFs. For instance, the three protein-interaction modules (BIOGRID-M392, BIOGRID-M698, BIOGRID-M170) are associated with mitochondrial genome maintenance and the TF motifs V$HNF1_C/V$HNF1_Q6 and V$GR_Q6/V$GR_Q6_01 are annotated to hepatocyte nuclear factor 1 homeobox A and glucocorticoid receptor, respectively.

The enrichment analysis in the cluster members of some p-metaomics models (Table S8) showed that the clustered markers are mainly governed by the same functional categories as their cluster centroids. For instance, the significant co-expression module LUAD-M722 associated with the digestive system process and the enriched co-expression module LUSC-M272 related to O-glycan processing are shared in eight of eleven cluster members of the p16-group (Table S8). In particular, the p58 cluster centroid, which represents the flatness shape radiomic feature, and the entire p58-cluster, which have shape-related members, engage exactly the same gene sets, implying that they are dominated by the same biological processes. This should be reasonably expected due to the inherent properties of the radiomic shape features.

Based on the above analysis, 13 disease-specific (12 LUAD, 1 LUSC) mRNA co-expression network modules were associated with enriched biological processes, such as the digestive system process, glycosphingolipid metabolic process, xenobiotic metabolic process, transcription initiation from RNA polymerase II promoter, and bicarbonate transport (Figure 8 and Figure S1). The inequality in LUSC versus LUAD modules probably reflects the inequality of our samples, as the majority are LUAD samples. Similarly, three protein-interaction modules were associated with the highly enriched process of mitochondrial genome maintenance. Furthermore, other biological processes and pathways, including O-glycan processing, glucosamine-containing compound catabolic process, glycosylation, regulation of hormone levels, melanocyte differentiation, muscle tissue morphogenesis, and DNA IR-DSBs and cellular response via ATM, were also ranked among the top 10 enriched terms identified by WebGestalt analysis (Figure 8 and Figures S1 and S2). Most of these processes have been implicated to a greater or lesser extent in lung cancer pathogenesis and metastasis [48,49,50,51,52,53,54,55,56,57,58,59].

*Xenobiotic metabolism* is of importance in tumors since biotransformation via the drug-metabolizing enzymes can result in either their activation or detoxification [60]. The lung, as a target organ for the toxicity of the inhaled compounds that are foreign to human life (xenobiotics), has a significant capability of biotransforming them to reduce potential toxicity [61]. Transcription plays a crucial role in a variety of cellular processes ranging from survival, cell growth, and differentiation. Malignant transformation is highly related to enhanced transcription of oncogenes, anti-apoptotic factors, and other transcription factors in cancer cells. Thereby, RNA polymerase II transcription is required to support the high demand of the transcripts, which is necessary for the maintenance of rapid growth and apoptosis resistance [51,62]. Glycosylation is an important enzymatic process that produces glycosidic linkages of saccharides to other saccharides, lipids, or proteins. It is involved in different aspects of cancer development, including cell–cell interactions, cell adhesion, malignant transformation, and metastasis [63,64]. Digestive process is a complex and important process of turning the food into nutrients, which the body uses for energy, growth, and cell repair. The digestive process was the most statistically significant enriched GO process identified in the gene sets of p-metaomics models, which is consistent with recent evidence on the emergence of tumor plasticity that mirrors the developmental history of organs [58]. This framework may also explain the presence of the other developmental-associated processes in our p-metaomics models, such as muscle tissue morphogenesis, positive regulation of axonogenesis, and melanocyte differentiation and regionalization. The latter was also found enriched in hypermethylated genes of NSCLC samples supporting in part a dedifferentiation cellular process in cancer [65]. Mitochondrial genome maintenance is understood as the mitochondrial DNA (mtDNA) replication and repair. To perform their functions, mitochondria carry their own genome; along with multiprotein machineries dedicated to maintaining the fidelity of genome replication, they promote transcription to spot and repair any DNA defects in retaining a low mutation rate in each cell generation. MtDNA, compared to nuclear DNA, possesses inadequate repair mechanisms and a high susceptibility to mutations, justifying its contribution to the development of cancer [66,67].

It is worth mentioning that the DNA IR-DSBs and cellular response via ATM, one of the most important findings according to 73 DEGs, demonstrated a strong statistical association with the highly coherent group of the p58-metaomics model. This association is induced by two genes involved in this pathway, namely, *FANCD2* and *NABP2* (hSSB1). *FANCD2* is implicated in the Fanconi anemia pathway, to orchestrate the maintenance of genome integrity and prevention from diseases including cancer. Currently different forms of *FANCD2* are reported to have an oncogenic or tumor suppressive role [68]. NABP2 protein is a guardian of genome stability and is a prognostic factor in NSCLC, according to a recent study [69,70].

In addition, the TFs’ target analysis by WebGestalt revealed four enriched predicted TFs, namely, HNF1A, ALX1, AR, and GR, and their target genes in seven p-metaomics gene sets (Figure 8 and Figure S2). HNF1A, ALX1, AR, and GR play certain roles in lung cancer development and progression, and/or they have been associated with the overall survival [71,72,73,74,75]. Similarly, the miRNAs’ target analysis by WebGestalt revealed four enriched predicted miRNAs, namely, miR-143, miR-17-3P, miR-380-3P, and miR-29A, miR-29B, miR-29C, and their target genes in five p-metaomics gene sets (Figure 8 and Figure S2), which have been reported to play tumor-suppressive, oncogenic, or regulatory roles in cancer including lung cancer [76,77,78,79].

#### 3.5.3. Evaluation of Significant Genes in Independent Lung Cancer Datasets

A further comparison with previously published work (curated transcriptomics lung adenocarcinoma datasets) that was conducted via CANCERTOOL revealed the high abundance of overlapping genes, and is presented as supplementary information (Tables S9 and S10). More precisely, all 73 DEGs were found differentially expressed in tumor versus healthy tissue, except 3 (Table S9). The most significant was *SPINK1* (9.949 × $10^{-35}$), which is in accordance with the study of Girard et al. [26]. Furthermore, 54 DEGs were found to be implicated in various progression stages of lung adenocarcinoma, while 41 and 46 DEGs were found to be different according to the *EGFR* or *KRAS* mutation status of the tumor, respectively (Table S10). Finally, 52, 45, and 8 DEGs were related to overall, disease-free, or metastasis-free survival, respectively (Table S10).

These findings not only emphasize the importance of the derived DEGs, but also support and justify their integration into p-metaomics models of lung cancer. The mixture of biological processes by the combination of transcriptomic markers inflicted by p-metaomics models reveal specific thematic patterns that may form hidden interconnections worthy of further experimental evaluation in diagnosis, prognosis, treatment, and survival analysis, via the examination of cost-effective and non-invasive imaging protocols. To this end, the value of such a joint exploration should be further evaluated in larger independent radiotranscriptomics datasets.

## 4. Discussion

This study provides a methodological approach to investigate radiotranscriptomics associations based on the concrete assumption that the phenotype of cancer is strongly dictated by its associated genetic origin. It has been widely considered that transcriptomic markers can achieve a certain degree of cancer predictability [3,80]. Complementing this set, radiomics markers can also provide predictive power, encoding certain tissue deformation patterns, leading to better and more precise diagnosis and treatment of cancer patients [4]. It is reasonable to assume, as in recent studies including ours, that the information encompassed by the late tissue deformation captured by imaging modalities might be largely explained by early genomic deregulations, as such alterations are caused by the genetic origin [3]. Thus, the information content of radiomics can be modeled as a homomorphic mapping guided by the transcriptomic basis. Since the cause (genomics) is expected to leave its footprint on the responsive effect (radiomics), a similar association is hidden in the markers measured by the corresponding modalities. Thus, the p-metaomics models obtained from transcriptomics regression to fit the radiomics markers could reveal the hidden biological substrate encoded in the anatomic imaging features, biologically justifying the use of radiomic markers.

The joint signature with measured radiomics and transcriptomics markers can be interpreted as complementing information from the two biological scales. Benefiting from the modeling of the transcriptomic markers from readily available radiomics markers in clinical practice, several studies have tried to elucidate associations between radiomics features and gene signatures for effective diagnosis and prognosis [81,82,83]. In our study, the modeling of “cheap” radiomics features with “expensive” transcriptomic markers was explored. The utility of such an approach is 2-fold. First, the joint signature entails pure transcriptomics markers and radiomics models, which form linear combinations of the former. Thus, it expands the original transcriptomics space with a more complex space spanned from linearly transformed dimensions, in a way similar to the transformed Principal Component Analysis (PCA) dimensions. However, the difference is that, instead of using the statistically significant dimensions in the distribution of data, the new space is formed by the dimensions that best characterize the radiomics features, preserving their anatomic content and structure. The second main utility of modeling is to give biological reasoning to the somewhat arbitrary concatenation of mass radiomic features. The transcriptomic modeling of such features reveals the coupling with certain transcriptomic markers, which can be readily associated with significant biological processes based on enrichment analysis. In this form, this modeling paves the ground for the biological justification of several arbitrarily introduced radiomics markers.

The transcriptomic signature, which was used to build the regression models of radiomics, was thoroughly examined for its significance in NSCLC, validating its ability to also differentiate between the malignant and control samples in several external datasets. Hence, a set of highly discriminative genes in NSCLC was thoroughly selected and used to produce the radiotranscriptomic models. The transcriptomics signature consists of 73 genes which are compared with the gene signatures/gene sets obtained from the used Datasets 2 and 4. This comparison showed an overlap of five genes (*ETV4* (tumor non-malignant signature), *STK32A*, *SPINK1*, *GOLT1A*, *KRT6A* (adenocarcinomas—squamous cell carcinomas signature)) in the first case, and an overlap in one (*KRT15*) of the three most prominent genes in the second case. The differences in the derived gene signature are related to the different methodology followed by these studies and our study to answer different research questions and scope. In many cases, a comparison with our study was not possible. For example, a comparison with signature/gene set of Dataset 3 was not considered useful because of the inclusion of only normal lung samples from this dataset. Moreover, the reported significant genes in the independent datasets could not be compared with our study, since these studies follow different research directions. More specifically, Dataset 5 was utilized by Wei et al. [29] to explore solely the statistically significant differences in *PRMT5* between the lung tissue paired (LUAD and adjacent normal) samples, Dataset 6 was used by Rousseaux et al. [31] to detect aberrant expressions of testis-specific/placenta-specific (TS/PS) genes in lung tumor samples, and Dataset 7 was used by Seo et al. [32] to identify somatic point mutations and transcriptional variants in lung adenocarcinoma. Lastly, a direct comparison with the basic radiotranscriptomic study of Nair et al. [15] was not possible due to the following main differences: (i) our signatures were extracted from microarray transcriptomic profiles of both patient samples and their non-malignant counterparts or control samples, and not only patient samples; (ii) our study focused on CT-extracted radiomic features, while Nair et al. [15] used the FDG PET/CT radiomics features.

The enrichment analysis on the significant genes involved in each p-metaomic model yields relevant biological support to the corresponding radiomics features. Each model with its biological origin concerns not only the central feature (p-metaomics) but also a variety of radiomic features in the same cluster, expressing the same distribution over the studied population. Through the coupling of genes in the p-metaomic models, our analysis highlights the significance of (a) *FANCD2*, *NABP2* (hSSB1), and *SPINK1* genes; (b) xenobiotic metabolism, bicarbonate transport, transcription, glycosylation, digestion, and developmental-related processes, as well as nuclear and mitochondrial DNA repair processes; and (c) TFs, such as HNF1, ALX1, AR, and GR, and (d) miR-143, miR-17-3P, miR-380-3P, and miR-29 family in NSCLC. Based on these observations, the p-metaomics models integrate quantitative radiomic features with thematic expression patterns, linking an important biological prospect to lung tumor characteristics. The presence of more than one thematic pattern in several p-metaomics models may reflect a multitude of alterations from a single biological or genomic etiology and forms a basis for further assumptions and experimental studies. Furthermore, the formation of p-metaomics models can motivate the design of novel imaging protocols that can specifically target radiotranscriptomic signatures [84]. The identified TFs and miRNAs and their target genes should also be further experimentally evaluated for their roles in the development and progression of NSCLC disease, considering that miRNAs and TFs’ targeting pathways provide potential candidates for therapeutic intervention. Similar observations regarding GO processes have been reported on the utilization of gene sets for the characterization of radiomic features through gene masking in NSCLC [85]. Smedley et al. [85] identified five of the aforementioned GO processes, namely, the developmental-related, post-translational and DNA repair-related, transport- and catabolism-related, hormone-related, and muscle-related GO processes, to predict radiomic features.

The histopathological features in the form of semantic features specified by the clinician could be associated with and related to the transcriptomic substructure of the tumor in NSCLC. Although this was not a primary research direction for this study, based on the results presented by Yip et al. [86], many of the radiomics clusters in this study, which can be regressed from the transcriptomics signature, can be associated with semantic features such as spiculation, contour, and texture. Furthermore, the mitochondrial dynamics and sphingolipid metabolic reprogramming, which are reflected in the p-78, p-83, and p-85 metaomics models, can indirectly relate to semantic textures [87,88].

This is a pilot study, which presents a methodological framework for the investigation of radiotranscriptomics correlations to reveal the underlying biological connection. However, this study has several limitations. A certain limitation is the small sample size of the simultaneously available microarray transcriptomics and imaging data, since only one dataset of a relatively small number of patients with both types of characteristic features was used. The absence of publicly available radiomics data combined with transcriptomics data restricts the capacity to investigate and further validate the significance of radiomic features as imaging-based biomarkers. The delivery of publicly available datasets with both radiomics and gene expression data for the same subjects is essential to facilitate research on radiotranscriptomics towards the development of robust models in cancer diagnosis and prognosis. Furthermore, the lack of imaging data from several clinical centers reduces the variability of the used data and does not reflect the heterogeneity of the patients with NSCLC among different centers. Additionally, there is not a standardization protocol for the extraction of the radiomic features, restricting the robustness and the reproducibility of radiomic features and making it difficult to compare the results with other studies.

Future research on the counterpart regression direction, investigating the modeling of the gene expression profiles from the easily and non-invasively captured radiomics measurements, will be performed. The tedious procedures of measuring mRNA through gene expression profiles could be avoided, when the radiomic features become robust biomarkers of the disease. Moreover, the analysis conducted via CANCERTOOL showed that a considerable number of DEGs play a role in overall survival, disease-free, or metastasis-free survival (Table S10). Since the p-metaomics features are derived by combinations of these DEGs and maintain the information of their transcriptomic substrate, the p-metaomics features may be correlated with survival. Hence, the correlation of the p-metaomics features with survival and other clinical variables will be investigated in a future study to further examine the clinical utility of these features. Furthermore, the collaboration with expert clinicians, who have the ability to perform the delineation of the tumor in the PET/CT examinations and extract SUV metrics, will be achieved in a future radiotranscriptomic study to incorporate this crucial examination into the study framework. Finally, expanding our formulation, more advanced models may be incorporated for inflicting the associations among radiomics and molecular markers, as it is dictated by the complexity of biological interactions in the diagnosis or prognosis pipeline.

## 5. Conclusions

This study proposes a methodological framework for the investigation of the associations between the radiomics and the transcriptomics in NSCLC to provide biological meaning of the radiomics features based on their modeling with a set of carefully selected transcriptomics markers. The genetic environment, as a cause of biological abnormalities related to the disease environment, would also dictate the phenotypic outcome reflected by the anatomic aberrations in radiological images. The proposed pipeline focuses on the identification of radiomic and transcriptomic markers, which are considered to be the most representative for lung cancer, resulting in a substantial reduction in the number of markers. The genes of the transcriptomics signature were thoroughly selected for their significant statistical correlation with radiomic features and their ability to discriminate malignant from non-malignant lung tissue, assisting in the diagnosis of the existence of lung cancer. The central radiomics features were simulated using regression models of these genes in order to enhance the relationship between the generative genomics data and the phenotypic outcome of the radiomics data. The discrimination ability of the simulated radiomics data (i.e., p-metaomics features) between malignant and non-malignant samples was also confirmed, showing their predictive power. Through the enrichment analysis of the genomic substrate of the p-metaomics features, a biological interpretation and support of the conceptual meaning of the radiomics were provided, revealing biological processes and pathways. The derived p-metaomics models provide a comprehensive approach for mapping the radiomic features with the biological information in NSCLC, and possibly in other cancer types, and can be expanded to answer multiple clinical problems related to cancer.

## Figures and Tables

**Table 1:** Overview of used datasets.

Datasets (GEO Accession)	Demographics and Clinical Characteristics	Radiomic Features	Datasets Utilization and Methodology
**Dataset 1** (GSE28827) **Samples** 24 NSCLC samples **No. of Genes** 24371 **Authors** Nair et al. (2012)	**Age, y (range)** 66.6 (46–84) **Gender(female)** 6 **Histology** 19 LUAD/5 LUSC **Stage** (4) (0) (5) IA1/(3) IA2/(6) IB (3) IIB/(3) IIIA **CT emphysema** 8	749 First-order statistics Shape 3D features GLCM GLRLM GLSZM GLDM NGTDM	Radiomics Features Extraction Meta-Radiomics Signature Extraction Radiotranscriptomics Cross-Correlation Analysis (SAM, Spearman rank correlation test, FDR 5%) p-metaomics Signature Modeling
**Dataset 2** (GSE75037) **Samples** 83 NSCLC samples 83 non-malignant lung samples **No. of Genes** 19227 **Authors** Girard et al. (2016)	**Age, y (range)** 68.1 (39–90) **Gender(female)** 59 **Histology** 83 LUAD **Stage** (24) IA/(26) IB (3) IIA/(17) IIB (1) III/(9) IIIA/(1) IIIB (2) IV **CT emphysema** n/a	-	Transcriptomics Signature Extraction (SAM, 2-fold change) Transcriptomics Validation p-metaomics Models Evaluation
**Dataset 3** (GSE76925) **Samples** 40 normal lung samples **No. of Genes** 17130 **Authors** Morrow et al. (2017)	**Age, y (range)** 65.7 (42–86) **Gender(female)** 25 **CT emphysema** 18	-	Transcriptomics Signature Extraction (SAM)
**Dataset 4** (GSE18842) **Samples** 44 NSCLC samples 44 non-malignant lung samples **Authors** Sanchez–Palencia et al. (2011)	**Age, y (range)** n/a **Gender** **(female)** n/a **Histology** 12 LUAD/32 LUSC **Stage** (6) IA/(32) IB (1) IIA/(2) IIB (2) IIIA/(1) IIIB **CT emphysema** n/a	-	Transcriptomics Validation (SVM classification, BHI and BSI calculation)
**Dataset 5** (GSE27262) **Samples** 25 NSCLC samples 25 non-malignant lung samples **Authors** Wei et al. (2012)	**Age, y (range)** 58.1 (34–77) **Gender(female)** n/a **Histology** 25 LUAD **Stage** (7) IA/(18) IB **CT emphysema** n/a	-	Transcriptomics Validation (SVM classification)
**Dataset 6** (GSE30219) **Samples** 143 NSCLC samples 14 normal lung samples **Authors** Rousseaux et al. (2013)	**Age, y (range)** 62.3 (44–84) **Gender(female)** 24 **Histology** 85 LUAD/58 LUSC **Stage** (117) IA/(12) IB (2) IIA/(7) IIB (3) IIIA/(2) IIIB **CT emphysema** n/a	-	Transcriptomics Validation (SVM classification)
**Dataset 7** (GSE40419-RNA-Seq) **Samples** 87 NSCLC samples 77 non-malignant lung samples **Authors** Seo et al. (2012)	**Age, y (range)** 63.8 (38–85) **Gender(female)** 34 **Histology** 87 LUAD **Stage** (31) IA/(24) IB (5) IIA/(8) IIB (10) IIIA/(3) IIIB (4) IV (2) n/a **CT emphysema** n/a	-	Transcriptomics Validation (SVM classification)

Abbreviations: GEO, Gene Expression Omnibus; NSCLC, non-small cell lung cancer; No, Number; LUAD, lung adenocarcinoma; LUSC, lung squamous cell carcinoma; y, years; CT, Computed Tomography; n/a, not available; GLCM, Gray Level Co-occurrence Matrix; GLRLM, Gray Level Run Length Matrix; GLSZM, Gray Level Size Zone Matrix; GLDM, Gray Level Dependence Matrix; NGTDM, Neighboring Gray Tone Difference Matrix; SAM, Significance Analysis of Microarrays; FDR, False Discovery Rate; SVM, Support Vector Machine; BHI, Biological Homogeneity Index; BSI, Biological Stability Index.

## Data Availability

The public radiotranscriptomics dataset can be freely accessed and downloaded at https://wiki.cancerimagingarchive.net/display/DOI/NSCLC+Radiogenomics%3A+Initial+Stanford+Study+of+26+Cases (accessed on 10 February 2020). The public transcriptomics datasets (accessed on 7 April 2020) can be downloaded at https://www.ncbi.nlm.nih.gov/geo/query/acc.cgi?acc=gse75037, https://www.ncbi.nlm.nih.gov/geo/query/acc.cgi?acc=GSE76925, https://www.ncbi.nlm.nih.gov/geo/query/acc.cgi?acc=GSE18842, https://www.ncbi.nlm.nih.gov/geo/query/acc.cgi?acc=GSE27262, https://www.ncbi.nlm.nih.gov/geo/query/acc.cgi?acc=GSE30219, https://www.ncbi.nlm.nih.gov/geo/query/acc.cgi?acc=GSE40419.

## Supplementary Materials

The following supporting information can be downloaded at: https://www.mdpi.com/article/10.3390/diagnostics13040738/s1, Table S1a: Overview of the used transcriptomics datasets in the study, Table S1b: Characteristics of patients and samples in Dataset 1 (GSE28827), Table S1c: Characteristics of patients and samples in Dataset 2 (GSE75037), Table S1d: Characteristics of healthy subjects and samples in Dataset 3 (GSE76925), Table S1e: Characteristics of samples in Dataset 4 (GSE18842), Table S1f: Characteristics of samples in Dataset 5 (GSE27262), Table S1g: Characteristics of samples in Dataset 6 (GSE30219), Table S1h: Characteristics of samples in Dataset 7 (GSE40419), Table S2: The 77 homogeneous clusters of radiomic features and their corresponding meta-radiomics features, Table S3: The 78 common differentially expressed genes (cDEGs), as extracted from the intersection of the 1st approach (Spearman + FDR across imaging features) and the 2nd approach (SAM), Table S4: Range of the values of all the validity metrics for the simulated p-metaomics features, Table S5: Values of all the validity metrics for the simulated features of the p-metaomics signature, Table S6a: Gene Ontology (GO) annotation in the category of biological process (noRedundant) of 73 cDEGs, Table S6b: Pathway annotation (KEGG, Panther, Wikipathway) of 73 cDEGs, Table S6c: Network annotation (TCGA RNA-Seq LUAD, TCGA RNA-Seq LUSC, PPI BIOGRID) of 73 cDEGs, Table S6d: Transcription factor (TF) and microRNA (miRNA) target annotation (MSigDB) of 73 cDEGs, Table S7: Overview of the most highly enriched biological terms across the 51 p-metaomics with their FDR values less than 0.70, Table S8: Overview of the most enriched biological terms across six (6) p-metaomics clusters with their FDR values, Table S9: Differential Expression Status of the 73 DEGs in well-known lung adenocarcinoma datasets, Table S10: Validation of the 73 DEGs in well-known lung adenocarcinoma datasets, Figure S1: Overview of the most highly enriched biological processes and pathways across 33 p-metaomics with their FDR values ≤ 0.1, and the set of genes involved, Figure S2: Overview of the most highly enriched transcription factors and microRNAs across 12 p-metaomics with their FDR values ≤ 0.1, and the set of target genes involved.
