# Supplementary material for: Synergies of Radiomics and Transcriptomics in Lung Cancer Diagnosis: A Pilot Study
Source: Diagnostics (Basel). 2023 Feb 15;13(4):738. doi: 10.3390/diagnostics13040738 (PMC9955510; doi:10.3390/diagnostics13040738)
Supplement: Supplementary file 1 [file diagnostics-13-00738-s001.zip › diagnostics-2218602-supplementary.pdf]

Supplementary Material

Synergies of Radiomics and Transcriptomics in Lung Cancer Diagnosis: A Pilot Study

Aikaterini Dovrou [1,†], Ekaterini Bei [1], Stelios Sfakianakis [2], Kostas Marias [2,3], Nickolas Papanikolaou [4] and Michalis Zervakis [1,*]

[1] Digital Image and Signal Processing Laboratory, School of Electrical and Computer Engineering (ECE), Technical University of Crete, GR-73100 Chania, Greece
[2] Computational BioMedicine Laboratory, Institute of Computer Science, Foundation for Research and Technology-Hellas, GR-70013 Heraklion, Greece
[3] Department of Electrical and Computer Engineering, Hellenic Mediterranean University, GR-70013 Heraklion, Greece
[4] Breast Unit, Champalimaud Clinical Centre, Champalimaud Foundation, Avenida Brasilia, 1400-038 Lisbon, Portugal
* Correspondence: mzervakis@tuc.gr; Tel.: +30-28210-37003
† Current address: Computational BioMedicine Laboratory, Institute of Computer Science, Foundation for Research and Technology-Hellas, GR-70013 Heraklion, Greece.

Section S1. Analytical Description of Radiomics Features Extraction

The radiomics features associated with the CT scans from dataset GSE28827 were extracted using the open-source python package pyradiomics [1]. This package requires the 3D - Region of interest (ROI) of the scan, which is the segmentation mask that indicates the pixel-based delineation of the tumor, for each patient. Scans with ROI < 10 pixels were excluded; thus 24 patients were used for further analysis. Subsequently, 749 CT radiomic features were extracted for the 24 patients, computed on the original images as well as on derived filtered images according to pyradiomics. In order to efficiently process the images, several filters were used including the Laplacian of Gaussian, Wavelet, Square, Square Root, Logarithm, Exponential and Gradient. Consequently, for each filtered and unfiltered image radiomics features were calculated related to the following categories:

(i) **First order statistics**: 10th Percentile, 90th Percentile, Energy, Entropy, Interquartile Range, Kurtosis, the minimum, the maximum, the median and the mean gray level intensity, Mean Absolute Deviation (MAD), Range, Robust Mean Absolute Deviation (rMAD), Root Mean Squared (RMS), Skewness, Total energy, Uniformity, and Variance. The first order statistics features describe the distribution of voxel intensities within the image ROI.

(ii) **Shape 3D features**: Elongation, Flatness, Least Axis Length, Major Axis Length, Maximum 2D Diameter (Column), Maximum 2D Diameter (Row), Maximum 2D Diameter Slice, Maximum 3D Diameter, Mesh Volume, Minor Axis Length, Sphericity, Surface Area, Surface Volume Ratio and Voxel Volume. The shape 3D features include descriptors of the three-dimensional (3D) size and shape of the ROI. They are independent from the gray level intensity distribution and thus they have the same values for all the original and the filtered images.

(iii) **Gray Level Co-occurrence Matrix (GLCM)**: Autocorrelation, Cluster Prominence, Cluster Shade, Cluster Tendency, Contrast, Correlation, Difference Average, Difference Entropy, Difference Variance, Inverse Difference (ID), Inverse Difference Moment Normalized (IDMN), Inverse Difference Normalized (IDN), Inverse Difference Moment (IDM), Informational Measure of Correlation (IMC) 1, Informational Measure of Correlation (IMC) 2, Inverse Variance, Joint Average, Joint Energy, Joint Entropy, Maximal Correlation Coefficient (MCC), Maximum Probability, Sum Average, Sum Entropy and Sum Squares. The GLCM features reflect contrast correlation and intensity cluster tendencies. The GLCM matrix contains the jointly probability occurrence of pairs of gray values along fixed axes within the image.

(iv) **Gray Level Size Zone Matrix (GLSZM)**: Gray Level Non uniformity, Gray Level Non uniformity Normalized, Gray Level Variance, High Gray Level Zone Emphasis, Large Area Emphasis, Large Area High Gray Level Emphasis, Large Area Low Gray Level Emphasis, Low Gray Level Zone Emphasis, Size Zone Non Uniformity, Size Zone Non Uniformity normalized, Small Area Emphasis, Small Area High Gray Level Emphasis, Small Area Low Gray Level Emphasis, Zone Entropy, Zone Percentage and Zone Variance. The GLSZM features express gray level Non-Uniformity and Gray Level Variance. The GLSZM quantifies gray level zones in the image, where the number of connected voxels that share the same gray level intensity constitutes a gray level zone.

(v) **Gray Level Run Length Matrix (GLRLM)**: Gray Level Non uniformity, Gray Level Non uniformity normalized, Gray Level Variance, High Gray Level Run Emphasis, Long Run Emphasis, Long Run High Gray Level Emphasis, Long Run Low Gray Level Emphasis, Low Gray Level Run Emphasis, Run Entropy, Run Length Non Uniformity, Run Length Non Uniformity normalized, Run Percentage, Run Variance, Short Run Emphasis, Short Run High Gray Level Emphasis and Short Run Low Gray Level Emphasis. The GLRLM matrix quantifies the size of consecutive pixels with the same gray-level intensity in a fixed direction and thus provide the size of homogeneous runs along specific axis for each gray level.

(vi) **Neighboring Gray Tone Difference Matrix (NGTDM)**: Busyness, Coarseness, Complexity, Contrast and Strength. The NGTDM quantifies the difference between a gray level intensity and the average gray level intensity of its neighbors.

(vii) **Gray Level Dependence Matrix (GLDM)**: Dependence Entropy, Dependence Non Uniformity, Dependence Non Uniformity normalized, Dependence Variance, Gray Level

Non Uniformity, Gray Level Variance, High Gray Level Emphasis, Large Dependence Emphasis, Large Dependence High Gray Level Emphasis, Large Dependence Low Gray Level Emphasis, Low Gray Level Emphasis, Small Dependence Emphasis, Small Dependence High Gray Level Emphasis and Small Dependence Low Gray Level Emphasis. The GLDM category quantifies gray level dependencies in an image.

Section S2. Analytical Results

Section S2.1. Transcriptomics Signature

The extraction of transcriptomics signature aims to identify the most discriminant and representative genes in lung cancer to be identified as transcriptomic markers.
SAM with 2-fold change identified 7014 significant genes with a q-value equal to 0%, using the gene expression profiles of the cancer samples of Dataset 2 and the normal samples of Dataset 3 (cancer *versus* control samples). The 5260 of these 7014 genes were declared as positive significant, indicating that their expression profiles are higher in cancer samples than in normal samples. Conversely, 1754 of the 7014 significant genes were identified as negative significant. Strengthening the discrimination power by testing on a different dataset, 2415 of the 7014 genes remain significant according to the 2-fold change within Dataset 2 (cancer *versus* control). 1573 genes from them were positive significant, while the rest 842 were negative significant. Since Dataset 1 engages both gene expression and radiomic features data for a cancer population, we identify the differentially expressed genes in this dataset to subsequently investigate the radiotranscriptomics correlations. Consequently, 2370 of the 2415 significant genes existed in patients of Dataset 1. From those, 1540 were positive significant, whereas the 830 were negative significant.

Section S2.1.1. Identification of strongly correlated genes with radiomic features

The statistical analysis of Spearman rank correlation test with FDR correction resulted in 6883 statistically significant correlations among pairs of differentially expressed genes and radiomic features. These statistically significant correlations refer to 95 different genes, as some genes are correlated with more than one imaging feature. To be more specific, 95 from the initial 2370 differentially expressed genes were correlated with at least one imaging feature.

A second statistical validation using SAM for quantitative problems revealed 651 statistically significant correlations between the differentially expressed genes and the radiomic features. Similar to the previous statistical test, some genes had significant correlations with more than one imaging feature. Thus, these 651 correlations referred to 137 significant genes.

With the Spearman rank correlation and the SAM for quantitative problem methods, the statistically significant associations were established between important genes with high differentiation ability and radiomic features. The statistical significance of these correlations was further validated with the extra criterion of FDR 5% in both cases. To strengthen the correlation between the two biological modalities, i.e. radiomics and transcriptomics markers, we combine the results of the two statistical tests by detecting and isolating their common genes for further analysis. There are 78 common genes, which have high discrimination ability and are significantly correlated with imaging features, as evaluated by two statistical methods. Thus, they form our transcriptomic signature showing high impact in the detection and diagnosis of lung cancer.

The Spearman rank correlation test measures the monotonic relationship between two variables and is a non-parametric test, which implies that it does not make any assumptions about the underlying distribution of the data. As the gene expression values and the radiomic feature values are not necessarily normally-distributed, the Spearman rank correlation test was preferred over the Pearson correlation test, which measures linear relationships between two variables. Hence, the quantitative radiomic and transcriptomic data were transformed into ranks to use the rank-based correlation test that can handle non-normal data and measure monotonic relationships. The ranks are important when we try to find the most important correlations rather than which variable is highly expressed. Since our aim was to identify the most significant correlations between genes and radiomic features, the rank-based tests were used.

Additional Files

Supplementary Table S1a: Overview of the used transcriptomics datasets in the study.

Supplementary Table S1b: Characteristics of patients and samples in Dataset 1 (GSE28827).

Supplementary Table S1c: Characteristics of patients and samples in Dataset 2 (GSE75037).

Supplementary Table S1d: Characteristics of healthy subjects and samples in Dataset 3 (GSE76925).

Supplementary Table S1e: Characteristics of samples in Dataset 4 (GSE18842).

Supplementary Table S1f: Characteristics of samples in Dataset 5 (GSE27262).

Supplementary Table S1g: Characteristics of samples in Dataset 6 (GSE30219).

Supplementary Table S1h: Characteristics of samples in Dataset 7 (GSE40419).

Supplementary Table S2: The 77 homogeneous clusters of radiomic features and their corresponding meta-radiomics features.

Supplementary Table S3: The 78 common differentially expressed genes (cDEGs), as extracted from the intersection of the 1st approach (Spearman+FDR across imaging features) and the 2nd approach (SAM).

Supplementary Table S4: Range of the values of all the validity metrics for the simulated p-metaomics features.

Supplementary Table S5: Values of all the validity metrics for the simulated features of the p-metaomics signature.

Supplementary Table S6a: Gene Ontology (GO) annotation in the category of biological process (noRedundant) of 73 cDEGs.

Supplementary Table S6b: Pathway annotation (KEGG, Panther, Wikipathway) of 73 cDEGs.

Supplementary Table S6c: Network annotation (TCGA RNA-Seq LUAD, TCGA RNA-Seq LUSC, PPI BIOGRID) of 73 cDEGs.

Supplementary Table S6d: Transcription factor (TF) and microRNA (miRNA) target annotation (MSigDB) of 73 cDEGs.

Supplementary Table S7: Overview of the most highly enriched biological terms across the 51 p-metaomics with their FDR values less than 0.7.

Supplementary Table S8: Overview of the most enriched biological terms across six (6) p-metaomics clusters with their FDR values.

Supplementary Table S9: Differential Expression Status of the 73 DEGs in well-known lung adenocarcinoma datasets.

Supplementary Table S10: Validation of the 73 DEGs in well-known lung adenocarcinoma datasets.

Supplementary Figure S1: Overview of the most highly enriched biological processes and pathways across 33 p-metaomics with their FDR values ≤ 0.1, and the set of genes involved.

Supplementary Figure S2: Overview of the most highly enriched transcription factors and microRNAs across 12 p-metaomics with their FDR values ≤ 0.1, and the set of target genes involved.

References

1. Van Griethuysen JJM, Fedorov A, Parmar C, Hosny A, Aucoin N, Narayan V, et al. Computational radiomics system to decode the radiographic phenotype. Cancer Res. 2017;77: e104–e107. doi:10.1158/0008-5472.CAN-17-0339
