# Supplementary figures and images for: Synergies of Radiomics and Transcriptomics in Lung Cancer Diagnosis: A Pilot Study
Source: Diagnostics (Basel). 2023 Feb 15;13(4):738. doi: 10.3390/diagnostics13040738 (PMC9955510; doi:10.3390/diagnostics13040738)

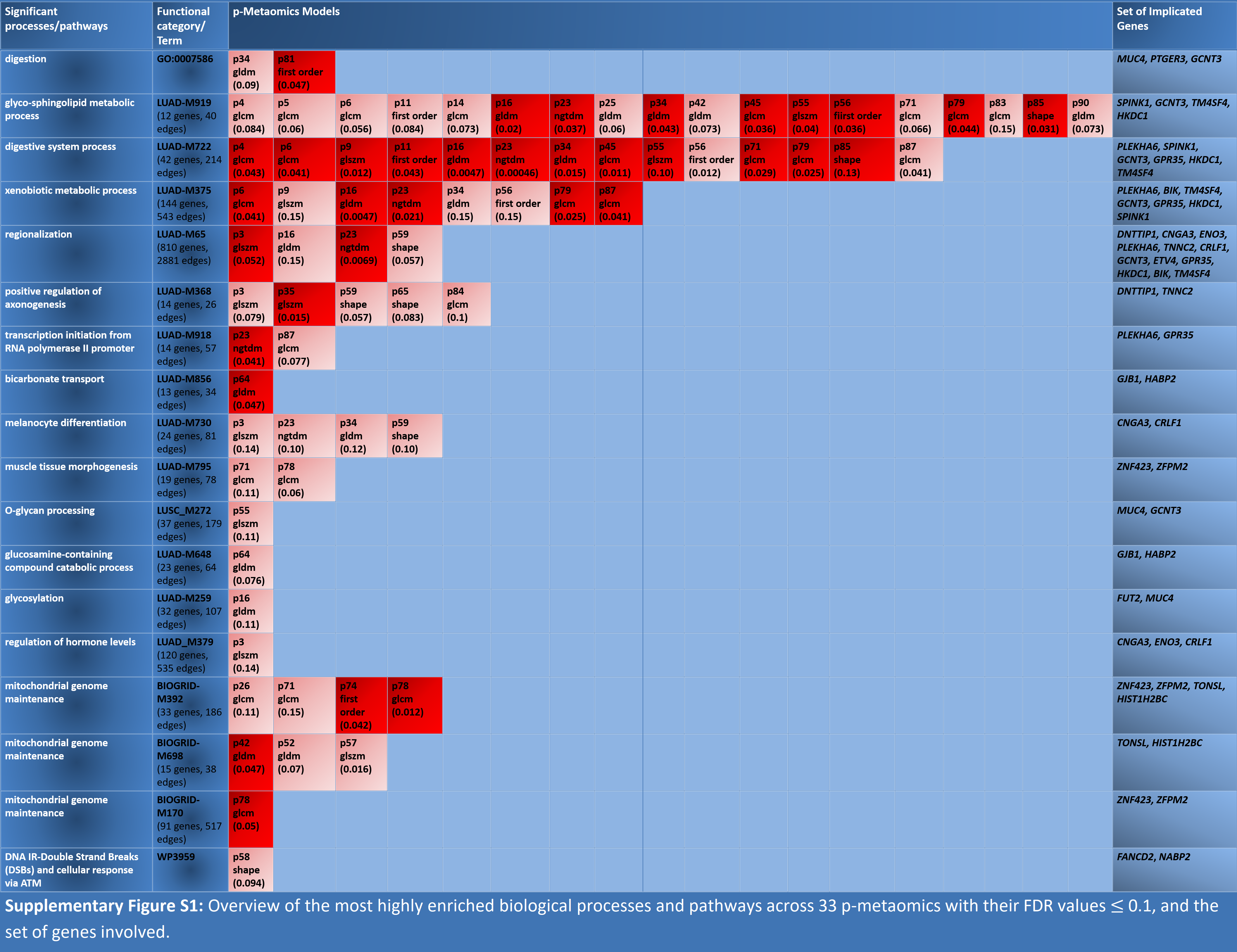

Supplement: Supplementary file 1 [file diagnostics-13-00738-s001.zip › Supplementary Figure S1.png]

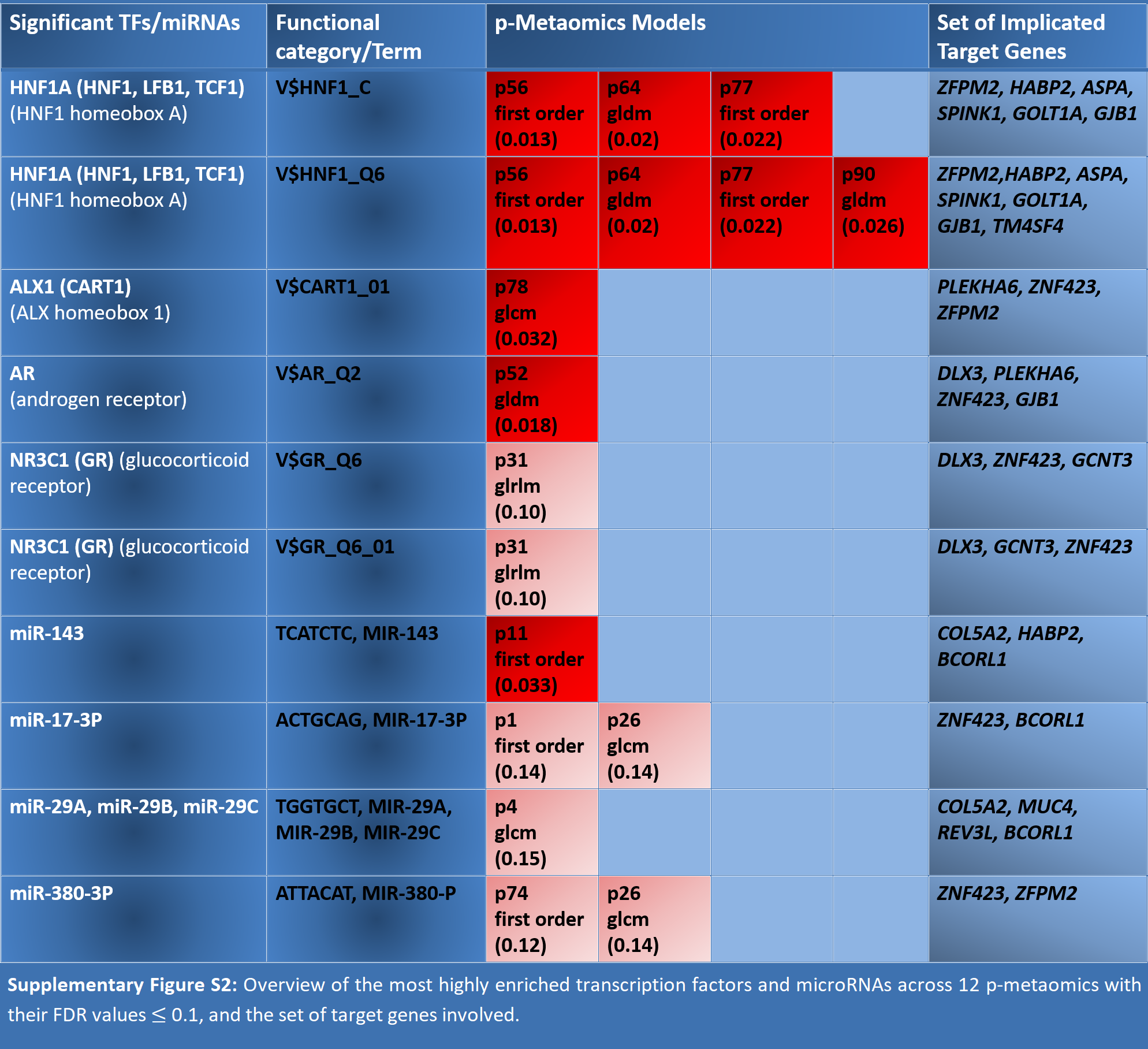

Supplement: Supplementary file 1 [file diagnostics-13-00738-s001.zip › Supplementary Figure S2.png]
